# Association of raisin and raisin‐containing food consumption with nutrient intake and diet quality in US children: NHANES 2001‐2012

**DOI:** 10.1002/fsn3.780

**Published:** 2018-10-15

**Authors:** Victor L. Fulgoni, James Painter, Arianna Carughi

**Affiliations:** ^1^ Nutrition Impact, LLC Battle Creek Michigan; ^2^ School of Public Health University of Texas Brownsville Texas; ^3^ Sun‐Maid Growers of California Kingsburg California

**Keywords:** children, healthy eating index, NHANES, nutrient intake, raisins

## Abstract

**Background:**

Raisins are a commonly consumed dried fruit and given their nutrient profile may offer nutritional and health benefits.

**Objective:**

To examine the association between consumption of raisins and raisin‐containing foods with nutrient intake and dietary quality in children.

**Methods:**

National Health and Nutrition Examination Survey (NHANES) data for 2001–2012 in those 2–18 years of age (*n* = 20,175) were used. Consumers of raisins (*n* = 154, 51.6% female) and raisin‐containing foods (*n* = 1,993, 52.5% female) were defined as reporting any consumption of raisins and raisin‐containing foods, respectively, during the first 24‐hr diet recall. Diet quality was assessed using the Healthy Eating Index (HEI)‐2010. Regression analyses were conducted comparing consumers and nonconsumers using appropriate sample weights and adjusted for demographic and lifestyle covariates with significance set at *p* < 0.01.

**Results:**

Regarding “nutrients of public health concern/shortfall nutrients” and “nutrients to limit,” raisin consumers had higher intakes of dietary fiber (23%), potassium (16%), magnesium (12%) with lower intakes of added sugars (−19%) than nonconsumers. Similarly, consumers of raisin‐containing foods also had higher intakes of dietary fiber (15%), potassium (5%), magnesium (11%), iron (6%), vitamin A (10%), and vitamin E (13%) and lower intake of sodium (−5%). Consumers of raisin and raisin‐containing foods had higher intakes of fruits (60%, 16%, respectively), whole fruits (119%, 23%, respectively) and whole grains (44%, 93%, respectively) and had a better diet quality as per higher total HEI 2010 scores (22%, 8%, respectively) than nonconsumers.

**Conclusion:**

In conclusion, consumption of raisins or raisin‐containing foods was associated with better nutrient intake and diet quality in American children.

## 1 INTRODUCTION

Increased intake of fruit and vegetables is an unequivocal recommendation by Dietary Guidelines worldwide. The Dietary Guidelines for Americans 2015–2020 (DGA) recommended adequate consumption of fruits and vegetables as part of healthy eating pattern [DHHS/USDA, 2015]. Dietary recommendations from the American Academy of Pediatrics for children and adolescents also include eating more fruits and vegetables and reducing added sugar [Gidding et al., 2006]. MyPlate (ChooseMyPlate.gov) recommends one to two cup equivalents of fruit per day for children depending on age, gender, and physical activity [USDA, 2010]. Despite these recommendations, about 25% of 1–3‐year‐old toddlers, about 40% of 4–8‐year‐old young children, 76% of 9–13‐year‐old children, and over 85% 14–18‐year‐old teens in the United States do not consume amount of fruit recommended [NCI, 2014a]. Fruits are naturally low in fat and sodium and can be important sources of potassium, dietary fiber, vitamin C, and folate.

Raisins (dried grapes) are a commonly consumed dried fruit [Keast, O'Neil, & Jones, 2011]. Raisins provide dietary fiber and important minerals and are low‐medium energy dense. A serving (43 g) of raisins provides 129 calories with 1.6 g dietary fiber, 0.2 g total fat, 25 g total sugar, 14 mg magnesium, 322 mg potassium, and 0.8 mg iron [USDA, 2016]. Raisins also contain various phytochemicals including flavonoids (catechins, quercetin, kaempferol, and rutin), hydrocinnamic acids (coutaric and cafterics), epicatechins, phytoestrogens (genestein and daidzein), and resveratrol [Karadeniz, Durst, & Wrolstad, 2000; Williamson & Carughi, 2010], but the physiological consequences of consumption of these compounds remain to be elucidated. However, intervention studies examining nutritional and health effect of raisin consumption have been mostly limited to adults [Williamson & Carughi, 2010; Anderson & Waters, 2013; Kanellos et al., 2014; Anderson, Weiter, Christian, Ritchey, & Bays, 2014; Bays, Weiter, & Anderson, 2015; Kanellos et al., 2013; Esfahani, Lam, & Kendall, 2014; ]. Satiating effects of raisins have been reported in a couple of studies with children [Patel, Luhovyy et al., 2013; Patel, Luhovyy, Mollard, Painter, & Anderson, 2013]. Using NHANES data, we have recently reported that raisin consumption in adults was associated with better diet quality, and better nutrient intake, with lower risks of having metabolic syndrome and being obese and (Fulgoni, Painter, & Carughi, 2017). Epidemiological studies evaluating the effect of raisins on diet quality and nutrient intake in children are very limited. In one cross‐sectional study in children aged 2–19 years, the intake of grapes and grape products (juice and raisins) was associated with a healthier overall dietary pattern and higher intake of certain nutrients [McGill, Keast, Painter, Romano, & Wightman, 2013]. The objective of this study was to evaluate the association of raisin or raisin‐containing foods consumption with nutrient intakes and diet quality in a large, nationally representative sample of US children.

## 2 METHODS

### 2.1 Subjects

Data from children age 2–18 years participating in the National Health and Nutrition Examination Survey (NHANES) 2001–2002, 2003–2004, 2005–2006, 2007–2008, 2009–2010, and 2011–2012 [CDC, 2015] were used for these analyses to improve sample size. Exclusion criteria included those with incomplete or unreliable 24‐hr recall data and those pregnant and/or lactating. Written informed consent was obtained for all participants or proxies (i.e., parents or guardians for children under 6 years; those 6–11 years had assistance from parents or guardians), and the survey protocol was approved by the Research Ethics Review Board at the NCHS.

### 2.2 Estimation of intake

Dietary intake data from the first 24‐hr recall dietary interviews collected using United States Department of Agriculture's (USDA) automated multiple‐pass method were utilized [Centers for Disease Control and Prevention (CDC), 2015]. Similar to our previous work (Fulgoni et al., 2017), intake of raisins were assessed using only two USDA food codes [USDA 2015a]: plain raisins (USDA food code 62125100) and cooked raisins (USDA food code 62125110). Intake of raisin‐containing foods was assessed using 147 USDA food codes that contained raisins as an ingredient. A list of top 40 foods containing raisins and their food codes is provided in Table 1. Consumers of raisins (*n* = 154) were defined as those consuming raisins in any amount during the 24‐hr recall. Consumers of raisin‐containing foods (*n* = 2,059) were defined as those consuming any quantity of foods that contained raisins during the 24‐hr recall. Energy and nutrient intake were determined similar to that of Fulgoni et al., 2017; in short, the respective Food & Nutrient Database for Dietary Studies for each NHANES cycle were used [USDA, 2015a]. MyPlate servings were calculated using the USDA MyPyramid Equivalents Database (MPED; for older surveys) and Food Patterns Equivalents Database (FPED; for more recent surveys) [Bowman, Friday, & Moshfegh, 2008; USDA, 2015b] which provides servings of major food groups and corresponding subgroups. The MyPlate food group intakes per day were calculated by aggregating the number of servings for all the foods reported in the 24‐hr recall.

**Table 1:** USDA food codes for top 40 raisin‐containing foods and their description [19]

Food Code	Description	Food Code	Description
56203030	Oatmeal, cooked, instant, fat not added in cooking	51180080	Bagel, with fruit other than raisins
51160110	Roll, sweet, cinnamon bun, frosted	51301800	Bagel, wheat, with raisins
62125100	Raisins	56203070	Oatmeal, cooked, instant, fat added in cooking
53542100	Granola bar, NFS	58116120	Empanada, Mexican turnover, filled with meat and vegetables
74406100	Steak sauce, tomato‐base	51161020	Roll, sweet, with fruit, frosted
57330000	Raisin Bran, Kellogg's	91739010	Raisins, chocolate covered
42501000	Nut mixture with dried fruit and seeds	52304010	Muffin, wheat bran
57329000	Raisin bran, NFS	53233040	Cookie, oatmeal, reduced fat, NS as to raisins
57227000	Granola, NFS	13210110	Pudding, bread
53241600	Cookie, butter or sugar, with fruit and/or nuts	51160100	Roll, sweet, cinnamon bun, no frosting
53510100	Danish pastry, with fruit	57000100	Oat cereal, NFS
51160000	Roll, sweet, no frosting	53610170	Coffee cake, crumb or quick‐bread type, with fruit
51129010	Bread, raisin	53102100	Cake or cupcake, applesauce, without icing or filling
51161000	Roll, sweet, with fruit, no frosting	27146160	Chicken with mole sauce
51180030	Bagel, with raisins	52404060	Bread, pumpkin
57331000	Raisin Bran, Post	53540000	Breakfast bar, NFS
13210410	Pudding, rice	57330010	Raisin Bran Crunch, Kellogg's
51129020	Bread, raisin, toasted	75141100	Cabbage salad or coleslaw with apples and/or raisins, with dressing
56203213	Oatmeal, cooked, instant, made with milk, fat not added in cooking	53237000	Cookie, raisin
28522000	Mole poblano (sauce)	51301120	Bread, wheat or cracked wheat, with raisins

### 2.3 Estimation of diet quality

USDA's Healthy Eating Index (HEI)‐2010 was used to estimate diet quality. HEI‐2010 has 12 components, and each component represents a particular aspect of diet quality [Guenther et al., 2013]. Dietary intake data (day 1) and the SAS code downloaded from the USDA website [NCI, 2014b] were used to determine HEI‐2010 scores.

### 2.4 Statistical analysis

SAS 9.2 (SAS Institute, Cary, NC) and SUDAAN 11 (RTI, Research Triangle Park, NC) were used for all analysis. The complex sample design of NHANES was considered using primary sampling units and strata with appropriate survey weights (day one dietary sample weights) in all intake analyses. Similar to our approach used previously (Fulgoni et al., 2017), least square means (LSMs) and standard errors (SEs) were generated via regression analyses for intakes of energy, nutrients, and food groups and diet quality in consumers and nonconsumers of raisins and raisin‐containing foods. Analyses of intakes of food groups and nutrient intakes were adjusted for age, ethnicity, gender, poverty income ratio, physical activity level, current smoking status, and energy intake. Analyses of energy intake and diet quality were adjusted for the same covariates but without energy intake; HEI scores are determined based on energy intake. For all analyses, *p* < 0.01 was deemed significant.

## 3 RESULTS

Approximately, 0.92% children (*n* = 154, 51.6% females) were consumers of raisins and 10.3% children (*n* = 1,993, 52.5% females) were consumers of raisin‐containing foods. Consumers of raisins as well as of raisin‐containing foods were younger than nonconsumers. Consumers of raisins were less likely to be Mexican American and smokers while the consumers of raisin‐containing foods were more likely to be non‐Hispanic White and less likely to be of other ethnicity (Table 2).

**Table 2:** Characteristics of children consumers of raisins and raisin‐containing foods, NHANES 2001–2012

Variables	Raisins			Raisins containing foods		
	NonConsumers Mean ± *SE*	Consumers Mean ± *SE*	*p* Value^a^	NonConsumers Mean ± *SE*	Consumers Mean ± *SE*	*p* Value^a^
Sample (*N*)	20,175	154		18,270	2,059	
Age (years)	10.1 ± 0.1	6.0 ± 0.6	**<0.0001**	10.1 ± 0.1	9.9 ± 0.2	0.2176
Gender						
Male (%)	50.7 ± 0.6	48.4 ± 5.6	0.6908	51.0 ± 0.6	47.5 ± 1.7	0.0438
Race/Ethnicity						
Mexican American (%)	13.5 ± 0.9	5.1 ± 1.4	**<0.0001**	13.6 ± 0.9	11.7 ± 0.9	0.1522
Other Hispanic (%)	6.1 ± 0.7	2.7 ± 1.1	0.0122	6.0 ± 0.7	5.9 ± 0.9	0.8936
Non‐Hispanic white (%)	59.1 ± 1.6	71.2 ± 4.8	0.0162	58.6 ± 1.6	65.0 ± 1.8	**0.0081**
Non‐Hispanic black (%)	14.4 ± 0.9	12.4 ± 3.4	0.5669	14.5 ± 0.9	13.2 ± 1.0	0.3570
Other (%)	6.9 ± 0.5	8.6 ± 3.1	0.5927	7.3 ± 0.5	4.2 ± 0.7	**0.0002**
Poverty income ratio	2.5 ± 0.04	2.8 ± 0.2	0.0854	2.5 ± 0.04	2.6 ± 0.07	0.0332
Smoker (%)	2.7 ± 0.3	0.0 ± 0.0	**<0.0001**	2.8 ± 0.3	2.0 ± 0.5	0.1651
Physical activity						
Sedentary (%)	12.6 ± 0.4	10.3 ± 2.3	0.3242	12.8 ± 0.4	10.7 ± 1.0	0.0491
Moderate (%)	19.9 ± 0.5	16.2 ± 3.8	0.3369	20.0 ± 0.6	19.2 ± 1.3	0.5946
Vigorous (%)	67.5 ± 0.6	73.5 ± 4.2	0.1601	67.3 ± 0.7	70.1 ± 1.6	0.1030

*Notes*

*SE*, standard error

^a^Comparison of consumers and nonconsumers.

Values in bold indicate significant at p<0.01.

Nutrient intakes differed significantly between the consumers of raisins and nonconsumers as well as between consumers and nonconsumers of raisin‐containing foods (Table 3). Consumers of raisins had significantly higher (*p* < 0.01) energy adjusted daily intakes of dietary fiber (22.9%), magnesium (11.6%) and potassium (16.0%), and lower intakes of added sugar (‐19.1%), total fat (−5.1%), and monounsaturated fat (−9.2%) compared to nonconsumers. Similarly, consumers of raisin‐containing foods also had significantly higher (*p* < 0.01) intakes of energy (10.8%), carbohydrate (1.8%), dietary fiber (14.6%), total sugar (3.2%), copper (7.9%), iron (6.6%), magnesium (11.5%), potassium (5.3%), vitamin A (10.0%), vitamin B6 (10.0%), and vitamin E (12.9%) with lower intakes of monounsaturated fat (−3.6%) and sodium (−4.6%) compared to nonconsumers. The intakes of other nutrients were not significantly different among consumers/nonconsumers of raisins as well as of consumers/nonconsumers raisin‐containing foods (Table 3).

**Table 3:** Energy and nutrient intakes in children consumers of raisins and raisin‐containing foods and nonconsumers: NHANES 2001–2012, gender combined data

Variables^a^	Raisins			Raisins containing foods		
	NonConsumers LSM ±* SE*	Consumers LSM ±* SE*	*p* Value^b^	NonConsumers LSM ±* SE*	Consumers LSM ±* SE*	*p* Value^b^
Energy (kcal)	1953 ± 12	1883 ± 50	0.1762	1934 ± 12	2142 ± 28	**<0.0001**
Protein (gm)	70.2 ± 0.4	73.0 ± 3.2	0.3723	70.2 ± 0.4	70.5 ± 0.8	0.6855
Carbohydrate (gm)	267 ± 1	278 ± 5	0.0330	267 ± 1	272 ± 1	**0.0030**
Dietary Fiber (gm)	13.3 ± 0.1	16.3 ± 0.8	**0.0002**	13.2 ± 0.1	15.1 ± 0.3	**<0.0001**
Total Sugars (gm)	132 ± 1	137 ± 5	0.3103	132 ± 1	136 ± 2	**0.0089**
Added Sugars (tsp)	20.0 ± 0.2	16.2 ± 1.1	**0.0004**	20.0 ± 0.2	19.7 ± 0.4	0.4645
Total Fat (gm)	72.9 ± 0.3	67.8 ± 1.7	**0.0035**	73.0 ± 0.3	71.6 ± 0.6	0.0177
MUFA (gm)	26.5 ± 0.1	24.1 ± 0.7	**0.0006**	26.6 ± 0.1	25.6 ± 0.3	**0.0002**
PUFA (gm)	14.7 ± 0.1	14.0 ± 0.4	0.0874	14.7 ± 0.1	15.2 ± 0.3	0.0830
SFA (gm)	25.5 ± 0.2	23.9 ± 0.9	0.0727	25.5 ± 0.2	24.8 ± 0.3	**0.0089**
Cholesterol (mg)	231 ± 3	218 ± 13	0.3231	232 ± 3	221 ± 6	0.0398
Calcium (mg)	985 ± 9	1070 ± 45	0.0573	987 ± 9	973 ± 19	0.4779
Copper (mg)	1.04 ± 0.02	1.22 ± 0.09	0.0357	1.03 ± 0.02	1.11 ± 0.03	**0.0001**
Iron (mg)	14.4 ± 0.1	15.5 ± 0.7	0.1434	14.3 ± 0.1	15.3 ± 0.3	**0.0005**
Magnesium (mg)	229 ± 1	256 ± 7	**0.0005**	227 ± 1	253 ± 4	**<0.0001**
Phosphorus (mg)	1237 ± 6	1307 ± 46	0.1323	1236 ± 6	1252 ± 14	0.2545
Potassium (mg)	2228 ± 15	2585 ± 108	**0.0012**	2220 ± 15	2337 ± 33	**0.0007**
Selenium (μg)	94.6 ± 0.6	97.7 ± 5.3	0.5657	94.5 ± 0.6	95.6 ± 1.4	0.4420
Sodium (mg)	3150 ± 18	3001 ± 130	0.2607	3161 ± 19	3016 ± 35	**0.0001**
Zinc (mg)	10.5 ± 0.2	11.0 ± 0.8	0.5434	10.6 ± 0.2	10.4 ± 0.3	0.4978
Vitamin A (μg)	563 ± 8	612 ± 37	0.1798	558 ± 8	614 ± 17	**0.0007**
Thiamin (mg)	1.55 ± 0.04	1.55 ± 0.05	0.8786	1.55 ± 0.04	1.59 ± 0.05	0.1568
Riboflavin (mg)	2.02 ± 0.04	2.07 ± 0.09	0.4945	2.01 ± 0.04	2.08 ± 0.05	0.0940
Niacin (mg)	20.8 ± 0.1	21.3 ± 1.3	0.7316	20.7 ± 0.2	21.7 ± 0.4	0.0356
Folate DFE (μg)	532 ± 5	545 ± 34	0.7094	531 ± 5	539 ± 12	0.5245
Vitamin B6 (mg)	1.69 ± 0.04	1.84 ± 0.13	0.2194	1.68 ± 0.04	1.85 ± 0.05	**0.0003**
Vitamin B12 (μg)	5.00 ± 0.13	5.19 ± 0.38	0.5848	5.00 ± 0.13	5.03 ± 0.16	0.7861
Vitamin C (mg)	89.4 ± 1.6	87.9 ± 7.6	0.8438	89.3 ± 1.7	89.8 ± 2.7	0.8593
Vitamin D (μg)	5.80 ± 0.07	5.70 ± 0.44	0.8214	5.79 ± 0.08	5.80 ± 0.16	0.9872
Vitamin E (mg)	5.96 ± 0.06	5.74 ± 0.23	0.3307	5.89 ± 0.06	6.65 ± 0.25	**0.0031**
Vitamin K (μg)	56.8 ± 1.2	50.8 ± 2.7	0.0196	56.7 ± 1.2	58.1 ± 2.3	0.4771

*Notes*

MUFA, monounsaturated fatty acids; PUFAs, polyunsaturated fatty acids; SFAs, saturated fatty acids; LSMs—least square means; *SE*, standard error.

^a^Values are adjusted for age, gender, ethnicity, poverty income ratio, physical activity level, current smoking status, and energy (except for energy); ^b^Comparison of consumers and nonconsumers.

Values in bold indicate significant at p<0.01.

Intake of raisins as well as of raisin‐containing foods was also associated with significant differences (*p* < 0.01) in food group intake (Table 4). Intakes of total fruit, whole fruit, and whole grain were significantly (higher *p* < 0.01) among consumers of raisins (60.1%, 119%, 44.1%, respectively) and raisin‐containing foods (15.6%, 22.7%, 93.2%, respectively) compared to nonconsumers. Overall diet quality (total scores for HEI‐2010) among consumers was significantly higher (21.5% for consumers of raisins and 7.7% for consumers of raisin‐containing foods) compared to nonconsumers (Table 5). HEI‐2010 subcomponent scores for total fruit (36.8%, 11.3%), whole fruit (81.4%, 19.6%), whole grain (60.0%, 81.9%), sodium (23.8%, 11.8%), and refined grains (22.7%, 7.9%) were higher for the consumers of raisins and consumers of raisin‐containing foods, respectively, compared to respective nonconsumers. Consumers of raisins also had a higher score for SoFAAS calories (30.8%), and consumers of raisin‐containing foods had a higher score for seafood and plant protein (22.2%) compared to their respective nonconsumers (Table 5).

**Table 4:** Intake of MyPlate food groups in children raisins and raisin‐containing foods consumers and nonconsumers: NHANES 2001–2012, gender combined data

Variables^a^	Raisins			Raisins containing foods		
	NonConsumers LSM ± *SE*	Consumers LSM ± *SE*	*p* Value^b^	NonConsumers LSM ± *SE*	Consumers LSM ± *SE*	*p* Value^b^
Total fruit (cup eq.)	1.1 ± 0.04	1.76 ± 0.18	**0.0002**	1.09 ± 0.04	1.26 ± 0.07	**0.0038**
Whole fruit (cup eq.)	0.56 ± 0.03	1.24 ± 0.15	**<0.0001**	0.56 ± 0.03	0.69 ± 0.06	**0.0032**
Total vegetable (cup eq.)	0.94 ± 0.03	0.99 ± 0.11	0.6426	0.94 ± 0.03	0.95 ± 0.05	0.9296
Total grain (oz eq.)	6.69 ± 0.13	6.48 ± 0.23	0.2653	6.69 ± 0.13	6.69 ± 0.19	0.9471
Whole grain (oz eq.)	0.53 ± 0.03	0.77 ± 0.10	**0.0095**	0.49 ± 0.03	0.96 ± 0.05	**<0.0001**
Total dairy (cup eq.)	2.10 ± 0.07	2.27 ± 0.16	0.2701	2.12 ± 0.07	1.99 ± 0.10	0.0362
Total protein group (oz eq.)	4.27 ± 0.12	4.32 ± 0.48	0.9178	4.26 ± 0.12	4.37 ± 0.18	0.3682

*Notes*

LSMs, least square means; SE, standard error

^a^Values are adjusted for age, gender, ethnicity, poverty income ratio, physical activity level, current smoking status, and energy; ^b^Comparison of consumers and nonconsumers.

Values in bold indicate significant at p<0.01.

**Table 5:** Healthy Eating Index (HEI)‐2010 total score and component scores of children raisins and raisin‐containing foods consumers and nonconsumers: NHANES 2001–2012, gender combined data

Variables^a^	Raisins			Raisins containing foods		
	NonConsumers LSM ± *SE*	Consumers LSM ± *SE*	*p* Value^b^	NonConsumers LSM ± *SE*	Consumers LSM ± *SE*	*p* Value^b^
HEI‐2010 Total score	45.7 ± 0.2	55.6 ± 1.7	**<0.0001**	45.5 ± 0.2	49.0 ± 0.5	**<0.0001**
Component 1 (Total vegetables)	2.16 ± 0.03	2.30 ± 0.22	0.5432	2.17 ± 0.03	2.15 ± 0.06	0.8378
Component 2 (Greens & Beans)	0.69 ± 0.03	0.58 ± 0.16	0.4424	0.69 ± 0.03	0.75 ± 0.05	0.2407
Component 3 (Total fruit)	2.52 ± 0.04	3.45 ± 0.20	**<0.0001**	2.50 ± 0.04	2.78 ± 0.08	**0.0010**
Component 4 (Whole fruit)	2.03 ± 0.04	3.68 ± 0.16	**<0.0001**	2.01 ± 0.04	2.40 ± 0.08	**<0.0001**
Component 5 (Whole Grains)	1.77 ± 0.04	2.83 ± 0.33	**0.0012**	1.66 ± 0.04	3.02 ± 0.13	**<0.0001**
Component 6 (Dairy)	6.73 ± 0.06	6.79 ± 0.32	0.8496	6.75 ± 0.06	6.55 ± 0.12	0.0949
Component 7 (Total Protein Foods)	3.63 ± 0.03	3.69 ± 0.15	0.7165	3.63 ± 0.03	3.68 ± 0.07	0.5114
Component 8 (Seafood & Plant Protein)	1.42 ± 0.04	1.94 ± 0.25	0.0404	1.40 ± 0.04	1.71 ± 0.07	**0.0008**
Component 9 (Fatty Acid Ratio)	3.91 ± 0.06	3.81 ± 0.37	0.7833	3.91 ± 0.06	3.94 ± 0.13	0.8601
Component 10 (Sodium)	4.94 ± 0.07	6.11 ± 0.44	**0.0084**	4.89 ± 0.07	5.47 ± 0.13	**<0.0001**
Component 11 (Refined Grains)	5.16 ± 0.07	6.33 ± 0.35	**0.0011**	5.13 ± 0.07	5.54 ± 0.15	**0.0074**
Component 12 (SoFAAS Calories)	10.7 ± 0.1	14.1 ± 0.8	**<0.0001**	10.8 ± 0.1	11.0 ± 0.3	0.3037

*Notes*

LSM, least square means; *SE*, standard error.

^a^Values are adjusted for age, gender, ethnicity, poverty income ratio, physical activity level, and current smoking status; ^b^Comparison of consumers and nonconsumers.

Values in bold indicate significant at p<0.01.

## 4 DISCUSSION

This is the first report to explore relationships of consumption of raisins and raisin‐containing foods with diet quality and nutrient intakes among US children using a large nationally representative sample. We combined data NHANES 2001–2012 thus providing a sample size of over 20,000 children in the present study. Approximately, 0.9% of the children in the United States reported consumption of raisins and 10% of US children reported consumption of raisin‐containing foods on the day of the recall and consumption of raisins and raisins containing foods was associated with better nutrient intake and diet quality.

Consumers of raisins had significantly higher intakes of energy adjusted dietary fiber, potassium and magnesium, and less added sugar than nonconsumers. Similarly, consumers of raisin‐containing foods consumed significantly more energy, dietary fiber, vitamin A, vitamin B6, vitamin E, iron, magnesium, and copper, with less sodium compared to nonconsumers. While consuming raisins and raisin‐containing foods was associated with numerous positive impacts on nutrient intake, the clinical significance of these changes was not assessed. Consumers of grapes and grape products (juice and raisins) using NHANES 2003–2008 [McGill et al., 2013] were reported to have higher intake of several key nutrients including dietary fiber, vitamin A, vitamin B6, vitamin C, calcium, potassium, and magnesium compared to nonconsumers. DGA has identified most of these nutrients (fiber, potassium, magnesium, and vitamin E) as—”shortfall nutrients” as these are currently underconsumed [DHHS/USDA, 2015]. Additionally, DGA has termed fiber and potassium as “nutrients of public health concern” as current intakes are low enough to possibly pose health risks [DHHS/USDA, 2015]. The intake of less than 5% of population is at or above the Adequate Intake for potassium or dietary fiber currently [DHHS/USDA, 2015]. Low intakes of potassium and dietary fiber are likely due to low intakes of fruits, vegetables, and whole grains [DHHS/USDA, 2015]. DGA recommended eating more fruits, vegetables, and whole grains along with low fat dairy to increase intake of potassium and fiber as well as other nutrients of public health concern [DHHS/USDA, 2015]. Consumers of raisins also had lower intakes of added sugars, and consumers of raisin‐containing foods had lower intakes of sodium compared to nonconsumers. DGA classified added sugar and sodium “nutrients to limit” and recommended eating patterns that are limited in added sugar and sodium [DHHS/USDA, 2015]. While sugars naturally occurring in raisins are not considered added sugars, any sugar added to the outside of raisins like that in certain ready‐to‐eat cereals would be considered added sugars.

The consumers of raisins and also of raisin‐containing foods also had higher intakes of fruits, whole fruit, and whole grain. The higher intake of these food groups is associated with healthier diets. HEI‐2010 is a validated measure of diet quality and is indicative of compliance with dietary recommendations. It has 12 components (nine components address adequacy intake and three address moderation of intake) each of which relate to a specific DGA recommendation [DHHS/USDA, 2011]. HEI has been previously used to assess whether diet quality changes over time [Juan, Guenther, & Kott, 2008], the effectiveness of dietary interventions, and to validate research tools and nutrition indices [Fulgoni, Keast, & Drewnowski, 2009]. It has also been effectively used to evaluate diets of population subgroups [Hiza, Casavale, Guenther, & Davis, 2013] and food environments [Reedy, Krebs‐Smith, & Bosire, 2010]. Several recent studies have also used HEI to assess relationships between intakes of nutrients, specific foods, and dietary patterns with health‐related outcomes [Fulgoni et al., 2017; Nicklas, O'Neil, & Fulgoni, 2012; Chiuve et al., 2012; Reedy et al., 2008; O'Neil, Nicklas, Rampersaud, & Fulgoni, 2011]. Total and many subcomponent HEI‐2010 scores of consumers of raisins and raisin‐containing foods were significantly higher than those of nonconsumers. Consumers of grapes and grape products (juice and raisins) were reported to have higher diet quality as compared to nonconsumers [McGill et al., 2013]. In adults, we previously found that HEI‐2010 total scores and scores of many subcomponents were higher in raisin consumers than nonconsumers [Fulgoni et al., 2017].

One limitation of this study is the use of cross‐sectional data, which cannot confer casualty. Additionally, given 24‐hr dietary recalls rely on participants’ memory to self‐report dietary intakes, they are subject to misreporting or reporting bias. Data used in this study were based on a single 24‐hr dietary recall and for some participants relied on intakes reported by parents/caregivers. Major strengths of this study included the use of a nationally representative large sample and the use of numerous covariates to adjust data in an attempt to remove potential confounding. However, residual confounding may still exist that could explain some of the results reported.

In conclusion, the results from this study suggest that consumption of raisins/raisin‐containing foods is associated with better diet quality and nutrient intake in American children.

## CONFLICT OF INTEREST

JP consults with Sun‐Maid Raisin Growers of California, the National Pasteurized Egg Board, Paramount Pistachios, and the National Dairy Council. AC works for Sun‐Maid Raisin Growers of California. VLF provides analyses of NHANES for members of the food/beverage industry.

## ETHICAL STATEMENTS

*Ethical review*: The Research Ethics Review Board at the National Center for Health Statistics of the Center for Disease Control and Prevention approved the survey protocol.

*Informed Consent*: Written informed consent was provided by all participants or proxies (i.e., parents or guardians).

## RESPONSIBILITIES

VLF and AC designed of the study. VLF conducted analysis; VLF, JP, and AC reviewed results. VLF developed the first draft of the manuscript; JP and AC provided edits. All authors read the final version.
